# Changes in retinal structural thickness after Lecanemab in Alzheimer's Disease

**DOI:** 10.1002/alz70856_101736

**Published:** 2025-12-25

**Authors:** Zhen Wang, Zhiming Pan

**Affiliations:** ^1^ Wenzhou Medical University, First Affiliated Hospital, Wenzhou, Zhejiang, China

## Abstract

**Background:**

The accumulation of soluble and insoluble aggregated amyloid‐beta (Aβ) may initiate or potentiate pathologic processes in Alzheimer's disease. Lecanemab, a humanized IgG1 monoclonal antibody that binds with high affinity to Aβ soluble protofibrils, is being tested in persons with early Alzheimer's disease (AD).

**Method:**

We conducted a one‐month single center phase 3 trial involving persons 50 to 90 years of age with early AD with evidence of amyloid on positron‐emission tomography (PET) or by cerebrospinal fluid testing. Participants were given intravenous lecanemab (10 mg per kilogram of body weight every 2 weeks). The primary end point was to evaluate the retinal structural thickness change from baseline at one month. Optical coherence tomography (OCT) was used to image and measure the retinal structural thicknesses [macula retinal nerve fiber layer (RNFL) and retinal ganglion cell complex (RGC)] in all participants. One eye (the eye with severe neurodegeneration) of each patient was included in the analysis.

**Result:**

A total of 8 participants were enrolled and assigned to receive lecanemab. The mean RNFL and RGC thickness was 38.16 (4.44) µm and 70.30 (3.93) µm respectively. A month of lecanemab, the mean RNFL and RGC thicknesses was 41.28 (5.13) µm and 70.61 (4.39) µm. The mean differences after a month of lecanemab were as follows: RNFL (difference, 3.11, *p* =  0.004) and RGC (difference, 0.31, *p* =  0.819).

**Conclusion:**

Lecanemab reduced markers of amyloid in early AD and resulted in significant thickening of the RNFL (axonal structure of the retina) and thickening of the RGC (neuronal structural associated with visual function in the retina. Longer trials are warranted to determine the efficacy and safety of lecanemab in eye of early AD patients.

